# Recruitment of Lyn from endomembranes to the plasma membrane through calcium-dependent cell-cell interactions upon polarization of inducible Lyn-expressing MDCK cells

**DOI:** 10.1038/s41598-017-00538-5

**Published:** 2017-03-28

**Authors:** Takao Morinaga, Sayuri Yanase, Aya Okamoto, Noritaka Yamaguchi, Naoto Yamaguchi

**Affiliations:** 1 Laboratory of Molecular Cell Biology, Graduate School of Pharmaceutical Sciences, Chiba University, Chiba, 260-8675 Japan; 2 Division of Pathology and Cell Therapy, Chiba Cancer Center Research Institute, Chiba, 260-8717 Japan

## Abstract

Src-family kinases, expressed in a wide variety of cell types, are anchored to cellular membranes through posttranslational lipid modifications and involved in diverse cellular signalling. In epithelial cells, Src-family kinases are localized at the plasma membrane and participate in epithelial functions. Epithelial cell polarity is achieved through dynamic reorganization of protein trafficking. To examine the trafficking of Src-family kinases between polarized and non-polarized epithelial cells, we generated an MDCK cell line that can inducibly express a protein of interest in a polarized state at any time. We show here that Lyn, a member of Src-family kinases, mainly localizes to the plasma membrane in polarized MDCK cells and to endomembranes in non-polarized MDCK cells. Cell-cell interactions between adjacent MDCK cells recruit Lyn from endomembranes to the plasma membrane even without cell attachment to extracellular matrix scaffolds, and loss of cell-cell interactions by calcium deprivation relocates Lyn from the plasma membrane to endomembranes through Rab11-mediated recycling. Therefore, using our MDCK cells expressing inducible Lyn, we reveal that calcium-dependent cell-cell interactions play a critical role in plasma membrane localization of Lyn in polarized MDCK cells.

## Introduction

Src-family non-receptor tyrosine kinases comprise at least eight members: c-Src, Lyn, c-Yes, Fyn, c-Fgr, Hck, Lck, and Blk. Src-family kinases consist of an N-terminal Src homology (SH) 4 domain that undergoes posttranslational lipid modification(s), an SH3 and an SH2 domains, a tyrosine kinase catalytic domain, and a C-terminal negative regulatory domain^1^. Src-family kinases are anchored to the cytoplasmic side of cellular membranes through posttranslational lipid modifications and are involved in transduction of tyrosine phosphorylation signals^2^.

Lyn, a member of Src-family kinases, is expressed in a wide variety of cell types, including epithelial cells, neuronal cells, and hematopoietic cells, and involved in diverse cellular signalling^3–6^. Following activation of receptors, such as glycosylphosphatidylinositol-anchored receptors, B-cell receptors, and integrins, Lyn is recruited to activated receptors at the plasma membrane and transduces signals downstream from the plasma membrane^5–7^. However, a considerable fraction of Lyn is found in intracellular compartments. Our previous studies revealed that newly synthesized Lyn traffics to the plasma membrane through the Golgi region^8^ and the palmitoylated SH4 domain is critical for the targeting of Lyn to the Golgi^9, 10^. Furthermore, we showed that cell detachment alters Lyn distribution in sucrose density-gradient fractionation in HeLa cells^11^.

Apical-basal cell polarity in epithelial cells arises through cell attachment to extracellular matrix scaffolds and cell-cell contacts between adjacent cells^12^. Polarized epithelial cells reorganize the molecular trafficking machinery to form asymmetric membrane domains and tight junctions^13, 14^. In polarized epithelial cells, Src-family kinases are involved in monolayer maintenance, vectorial vesicular transport, and tight junction formation^15–17^. Although Src-family kinases, including Lyn, are known to localize predominantly to the plasma membrane in polarized epithelial cells^3^, it remains to be elucidated whether establishment of cell polarity affects the trafficking pathway of Src-family kinases.

In this study, we generated Madin-Darby canine kidney (MDCK) cell lines inducibly expressing Src-family kinases and examined the localization of Lyn in the different culture conditions. We found that MDCK cells are capable of localizing Lyn mainly to the plasma membrane in polarized conditions and to endomembranes in non-polarized conditions. Upon depolarization, Lyn is translocated from the plasma membrane to endomembranes in a manner dependent on Rab11 activity. Moreover, the localization of Lyn at the plasma membrane depends on calcium-dependent cell-cell interactions irrespective of cell-scaffold interactions.

## Results

### Generation of an MDCK cell line expressing inducible Lyn

**Figure 1:**
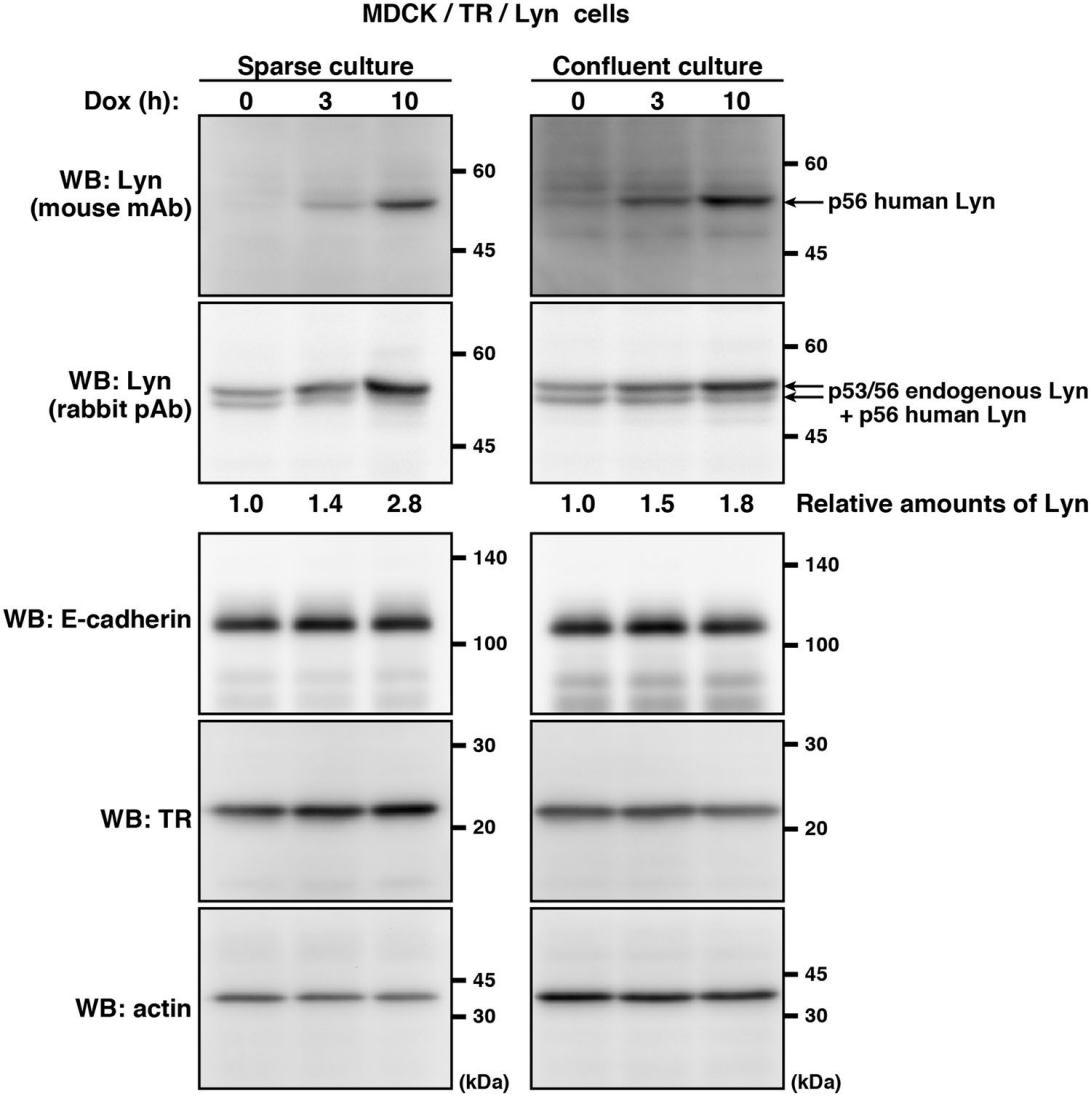
Establishment of an MDCK cell line expressing inducible Lyn. MDCK/TR/Lyn cells were grown in sparse and confluent cultures, which generates non-polarized cells and polarized cells, respectively. The cells were subsequently treated with 1 μg/ml doxycycline (Dox) for the indicated times. Whole cell lysates were subjected to Western blot analysis using the indicated antibodies. TR, tetracycline repressor. Molecular size markers are shown in kDa. The amount of p53/p56 Lyn in Dox-treated cells is expressed as the value relative to that in cells without Dox treatment after normalization with actin levels. For better clarity and conciseness of the presentation, cropped blots are shown. The full-length blots are presented in Supplementary Fig. S6.

**Figure 2:**
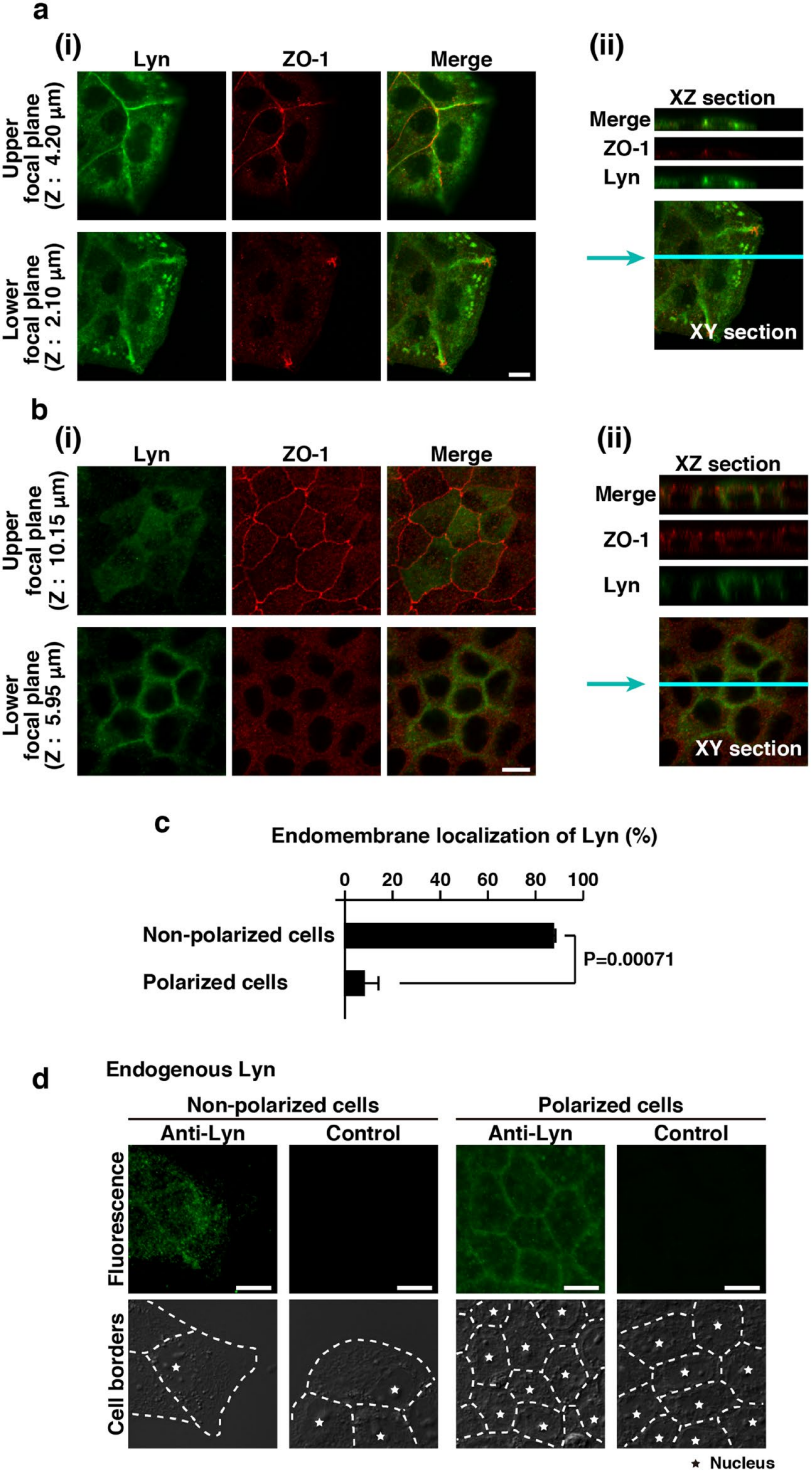
Localizations of Lyn in MDCK cells under the different culture conditions. **(a**–**c)** MDCK/TR/Lyn cells grown in sparse (non-polarized) **(a)** or confluent (polarized) (**b**) culture were treated with 1 μg/ml Dox for 10 h. Cells were fixed and then stained with rabbit polyclonal anti-Lyn antibody (green) and anti-ZO-1 antibody (red). The positions of the focal plane (Upper and Lower) are indicated along the z-axis. Representative XY sections (**i**) and XZ sections on the blue line **(ii)** are shown. Scale bar, 10 μm. **(c)** The number of cells in which Lyn was appreciably accumulated to endomembranes was counted. Cells grown at the cell periphery of each colony were regarded as non-polarized cells. The data represent the mean ratio ± S.D. from three independent experiments in which at least 50 cells for each category were scored. The significant difference is calculated by one-tailed Student’s *t* test. (**d**) Parental MDCK cells were grown in sparse (non-polarized) or confluent (polarized) culture. Cells were fixed and stained with rabbit polyclonal anti-Lyn antibody. Control samples were stained with secondary antibody alone. For eye guide, cell borders and nuclei are marked by dotted lines and stars, respectively. Scale bar, 10 μm.

Madin-Darby canine kidney (MDCK) cells cultured as confluent monolayers acquire apical-basal cell polarity. Because MDCK cells grown in confluent culture conditions can be hardly transfected with expression vectors, we generated an MDCK cell line expressing tetracycline-inducible human Lyn (MDCK/TR/Lyn). Fortuitously, mouse monoclonal anti-Lyn antibody (mouse mAb) was found to react to inducible human Lyn (the 56-kDa isoform) but not endogenous canine Lyn (two isoforms at 56 and 53 kDa), whereas rabbit polyclonal anti-Lyn antibody (rabbit pAb) is capable of reacting to both canine and human Lyn (Supplementary Fig. S1). Western blotting analysis showed that treatment of MDCK/TR/Lyn cells with doxycycline (Dox), a tetracycline derivative, induces expression of human Lyn (approximately 2~3-fold over endogenous Lyn), whose expression is repressed before Dox treatment (Fig. 1). In other words, this cell line has the advantage of no expression leakage of human Lyn unless Dox is added. Inducibly expressed Lyn was easily detected as early as 3 h after Dox treatment, irrespective of culture conditions (Fig. 1). Although v-Src, a constitutively active form of its cellular counterpart c-Src, is capable of degrading the adhesion molecule E-cadherin and destroying MDCK cell monolayers^15, 18^, inducible expression of Lyn in MDCK/TR/Lyn cells did not cause E-cadherin degradation and thereby preserving MDCK cell monolayers (Figs 1 and 2a,b; Supplementary Fig. S2). These results indicate that, upon Dox addition, human Lyn is capable of being synchronously expressed in most of confluent MDCK monolayer cells.

### Localizations of Lyn in MDCK cells under the different culture conditions

Apical-basal cell polarity is required for the formation and maintenance of tight junctions^19^. The tight junction protein ZO-1 lined up at the upper area of cell-cell contact sites in confluently cultured MDCK/TR/Lyn cells (Fig. 2b). The lining of ZO-1 was not disturbed upon inducible expression of Lyn (Supplementary Fig. S2), whereas ZO-1 was scattered at the plasma membrane in sparsely cultured MDCK/TR/Lyn cells (Fig. 2a). These results confirmed that MDCK/TR/Lyn cells cultured as confluent monolayers acquire apical-basal cell polarity. In MDCK/TR/Lyn cells cultured to confluence (polarized cells), the majority of inducibly expressed Lyn were localized at the plasma membrane (Fig. 2b,c). However, in MDCK/TR/Lyn cells cultured sparsely (non-polarized cells), inducibly expressed Lyn was obviously accumulated to punctate endomembrane structures other than the plasma membrane (Fig. 2a,c). Similar to inducibly expressed Lyn in MDCK/TR/Lyn cells, endogenous Lyn in parental MDCK cells was localized at the plasma membrane in a polarized state and distributed to endomembranes and the plasma membrane in a non-polarized state (Fig. 2d), which was confirmed by knockdown of endogenous Lyn (Supplementary Fig. S3). These results suggest that differential localizations of Lyn to endomembranes and the plasma membrane in MDCK cells may be regulated by non-polarized and polarized states, respectively.

### Differential localizations of Lyn in non-polarized and polarized MDCK cells

**Figure 3:**
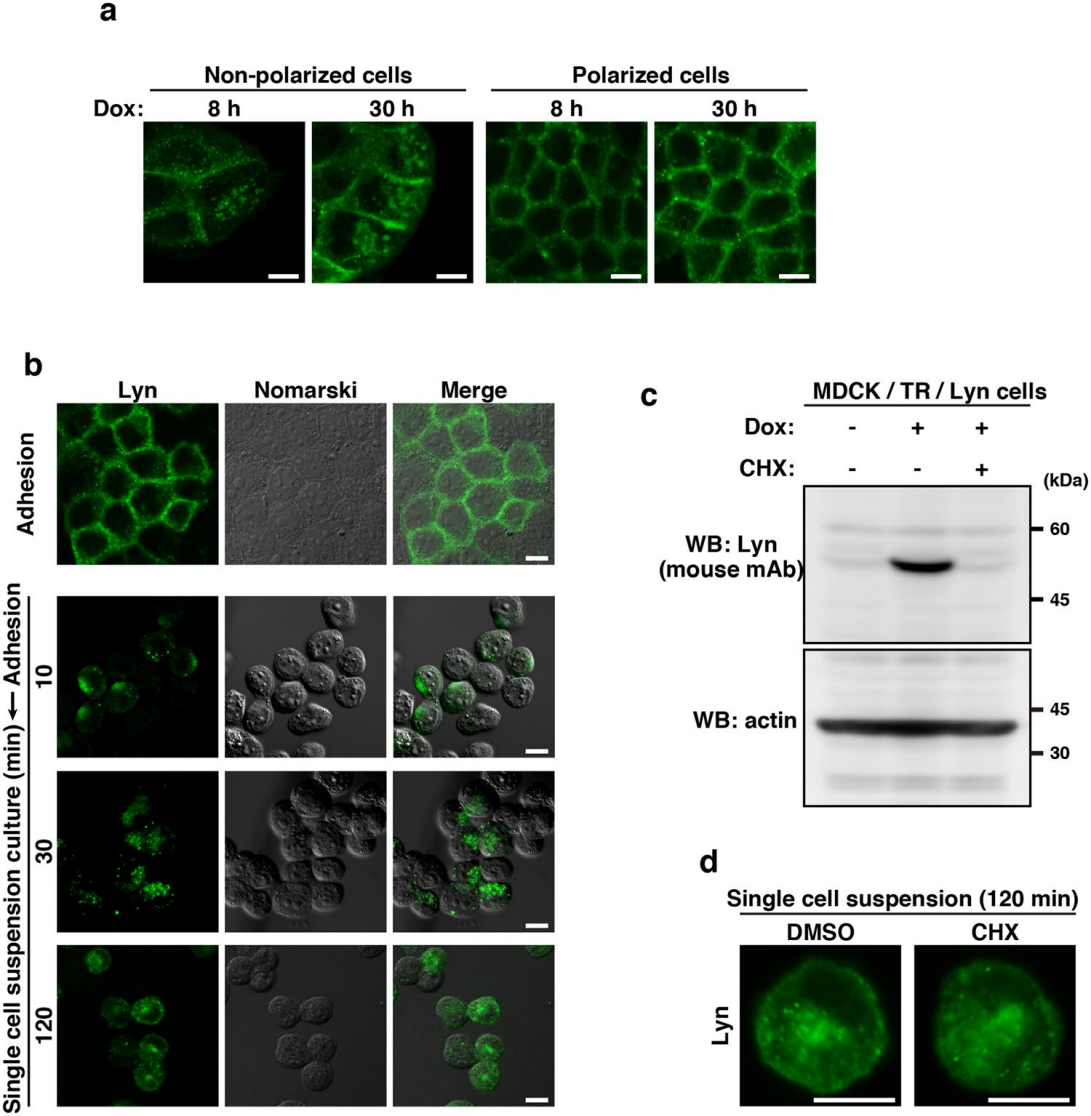
Differential localizations of Lyn in non-polarized and polarized MDCK cells. **(a**,**b**,**d)** Immunofluorescence staining was performed using mouse monoclonal anti-Lyn antibody (green). Scale bars, 10 μm. **(a)** MDCK/TR/Lyn cells were cultured in sparse or confluent conditions and then treated with 1 μg/ml Dox for 8 h and 30 h. **(b)** MDCK/TR/Lyn cells cultured to confluence (polarized cells) were treated with 1 μg/ml Dox for 8 h (Adhesion), detached with trypsin treatment and subsequently cultured for the indicated time (min) in a spinner flask with calcium- and serum-free IMDM containing 1 μg/ml Dox (Adhesion → Single cell suspension culture). (**c**) MDCK/TR/Lyn cells cultured to confluence (polarized cells) were treated with 1 μg/ml Dox in the presence or absence of 200 μg/ml cycloheximide (CHX) for 120 min. Whole cell lysates were subjected to Western blot analysis using the indicated antibodies. Molecular size markers are shown in kDa. The full-length blots are presented in Supplementary Fig. S7. **(d)** MDCK/TR/Lyn cells cultured to confluence (polarized cells) were treated with 1 μg/ml Dox for 10 h, detached with trypsin treatment and subsequently cultured for 120 min in a spinner flask with calcium- and serum-free IMDM (single cell suspension cultures) in the presence or absence of 200 μg/ml CHX for 120 min.

Our studies have shown that newly synthesized Lyn is first accumulated to perinuclear endomembranes, including the Golgi pool of caveolin, and then transported to the plasma membrane in COS-1 and HeLa cells that are cultured sparsely^8, 11, 20, 21^. We then examined whether the localization of Lyn might change from endomembranes to the plasma membrane in non-polarized MDCK cells with time after Dox treatment. During 8 h of Dox treatment, newly synthesized Lyn was accumulated to endomembranes in non-polarized MDCK cells, despite the presence of newly synthesized Lyn at the plasma membrane in polarized MDCK cells (Fig. 3a). These two contrasting patterns of Lyn localization in non-polarized and polarized MDCK cells were sustained at least 30 h after Dox treatment (Fig. 3a). To examine whether depolarization of MDCK cells could affect the localization of Lyn, Lyn was inducibly expressed in polarized MDCK cells followed by trypsinization and a subsequent spinner flask culture. The resulting cells became round in shape and eventually lost their polarity during a single cell suspension culture. Notably, Lyn became accumulated to endomembranes after 2 h of a single cell suspension culture in a spinner flask (Fig. 3b). These results suggest that endomembrane localization of Lyn is induced by a non-polarized cell state but plasma membrane-localization of Lyn is promoted by a polarized cell state.

Next, to examine whether Lyn accumulated to endomembranes during a single cell suspension culture was transported from the plasma membrane, we used cycloheximide (CHX), a protein synthesis inhibitor. Western blotting analysis showed that inducible expression of Lyn in MDCK/TR/Lyn cells was completely blocked by treatment with CHX for at least 2 h (Fig. 3c). Polarized MDCK/TR/Lyn cells expressing inducible Lyn at the plasma membrane for 10 h were detached and cultured for 2 h in a single cell suspension culture in the presence of CHX or dimethyl sulfoxide (DMSO, a solvent control). Although inhibition of protein synthesis by CHX seemed to slightly decrease the level of plasma membrane-localized Lyn upon cell depolarization (compare DMSO with CHX in Fig. 3d), Lyn became accumulated to endomembranes in MDCK/TR/Lyn cells that lost their polarity in the presence of CHX (Fig. 3d). These results suggest that depolarization of MDCK cells induces the translocation of Lyn from the plasma membrane to endomembranes.

### Translocation of Lyn to endomembranes through Rab11-positive endosomes

**Figure 4:**
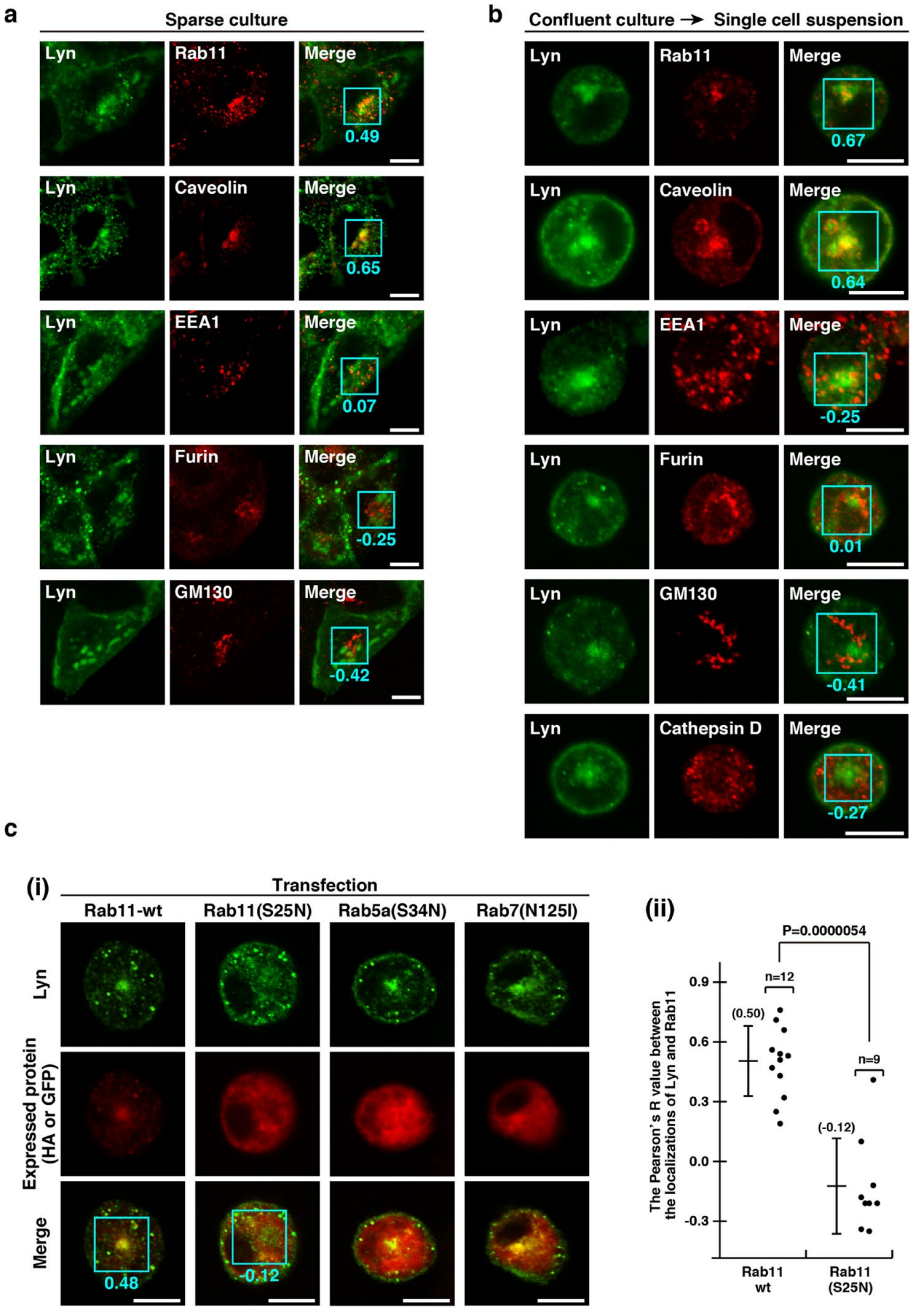
Endomembrane localization of Lyn through Rab11-positive endosomes. **(a**,**b)** Double immunofluorescence staining was performed using mouse monoclonal or rabbit polyclonal anti-Lyn antibody (green) together with the indicated antibody (red). The Pearson’s R value determined from the green and red signals in the region of interest (ROI; blue square) is shown in each image. Scale bars, 10 μm. **(a)** MDCK/TR/Lyn cells sparsely cultured (non-polarized cells) were treated with 1 μg/ml Dox for 10 h. **(b)** MDCK/TR/Lyn cells cultured to confluence (polarized cells) were treated with 1 μg/ml Dox for 8 h, detached with trypsin and subsequently cultured in a spinner flask with calcium- and serum-free IMDM containing 1 μg/ml Dox for 2 h. **(c)** MDCK/TR/Lyn cells transiently transfected with the indicated plasmid were cultured to confluence and then treated with 1 μg/ml Dox for 8 h, detached with trypsin and subsequently cultured in a spinner flask with calcium- and serum-free IMDM for 2 h. **(i)** HA-tagged Rab11-wt (wild-type Rab11) and HA-tagged Rab11(S25N) (dominant-negative Rab11) were stained with rabbit anti-HA antibody (red). The images of GFP-conjugated Rab5a(S34N) (dominant-negative Rab5) and GFP-conjugated Rab7(N125I) (dominant-negative Rab7) were pseudocoloured in red. Lyn was stained with mouse monoclonal anti-Lyn antibody (green). The Pearson’s R value determined from the green and red signals in the ROI (blue square) is shown in each representative image of Rab11- or Rab11(S25N)-expressing cell. Scale bars, 10 μm. **(ii**) The plots represent the Pearson’s R values determined from Lyn and Rab11-wt or Lyn and Rab11(S25N) in the ROI in each cell. n, cell number.

To characterize the endomembrane compartment that harbours Lyn, we compared the staining patterns of Lyn with those of several marker proteins, such as the recycling endosome proteins Rab11 and caveolin, the early endosome protein EEA1, the *trans*-Golgi protein furin, the *cis*-Golgi protein GM130, and the lysosome protein cathepsin D. The levels of co-localization of Lyn and other organelle markers were examined by determining the Pearson’s R value. Among these, Rab11 and caveolin were appreciably co-localized with Lyn in sparsely cultured, non-polarized MDCK/TR/Lyn cells, although none of them was perfectly co-localized with Lyn (Fig. 4a). To further examine the endomembrane localization of Lyn, we compared the localization of Lyn with that of the organelle markers when polarized MDCK cells were detached and subsequently cultured in single cell suspension as described in Fig. 3b. Whereas Lyn was localized largely to the plasma membrane in confluent, polarized MDCK/TR/Lyn cells (see also Fig. 2b), Lyn became appreciably co-localized with Rab11 or caveolin after confluent, polarized MDCK/TR/Lyn cells were cultured in single cell suspension (Fig. 4b). These results suggest that depolarization by single cell suspension changes the localization of Lyn from the plasma membrane to endomembranes that include Rab11- or caveolin-positive recycling endosomes.

Since Rab11 serves as an effector for various membrane trafficking pathways^14, 22–24^, we examined whether accumulation of Lyn to endomembranes was regulated by Rab11 GTPase activity. Notably, overexpression of dominant-negative Rab11(S25N) (inactive form) dispersed Lyn from the perinuclear region to the cell periphery in a single cell suspension culture, whereas overexpression of wild-type Rab11, dominant-negative Rab5a(S34N), or dominant-negative Rab7(N125I) did not interfere with perinuclear accumulation of Lyn upon depolarization (Fig. 4c). These results suggest that active Rab11 but not Rab5 nor Rab7 is required for perinuclear accumulation of Lyn, implicating that the active Rab11 may directly associates with Lyn or indirectly interact with Lyn-containing endomembranes through Rab11-positive endomembranes. At present, we hypothesize that Rab11-mediated endosomal trafficking plays a regulatory role in Lyn trafficking during depolarization of MDCK cells.

### Role for palmitoylation of Lyn in its endomembrane localization in MDCK cells

**Figure 5:**
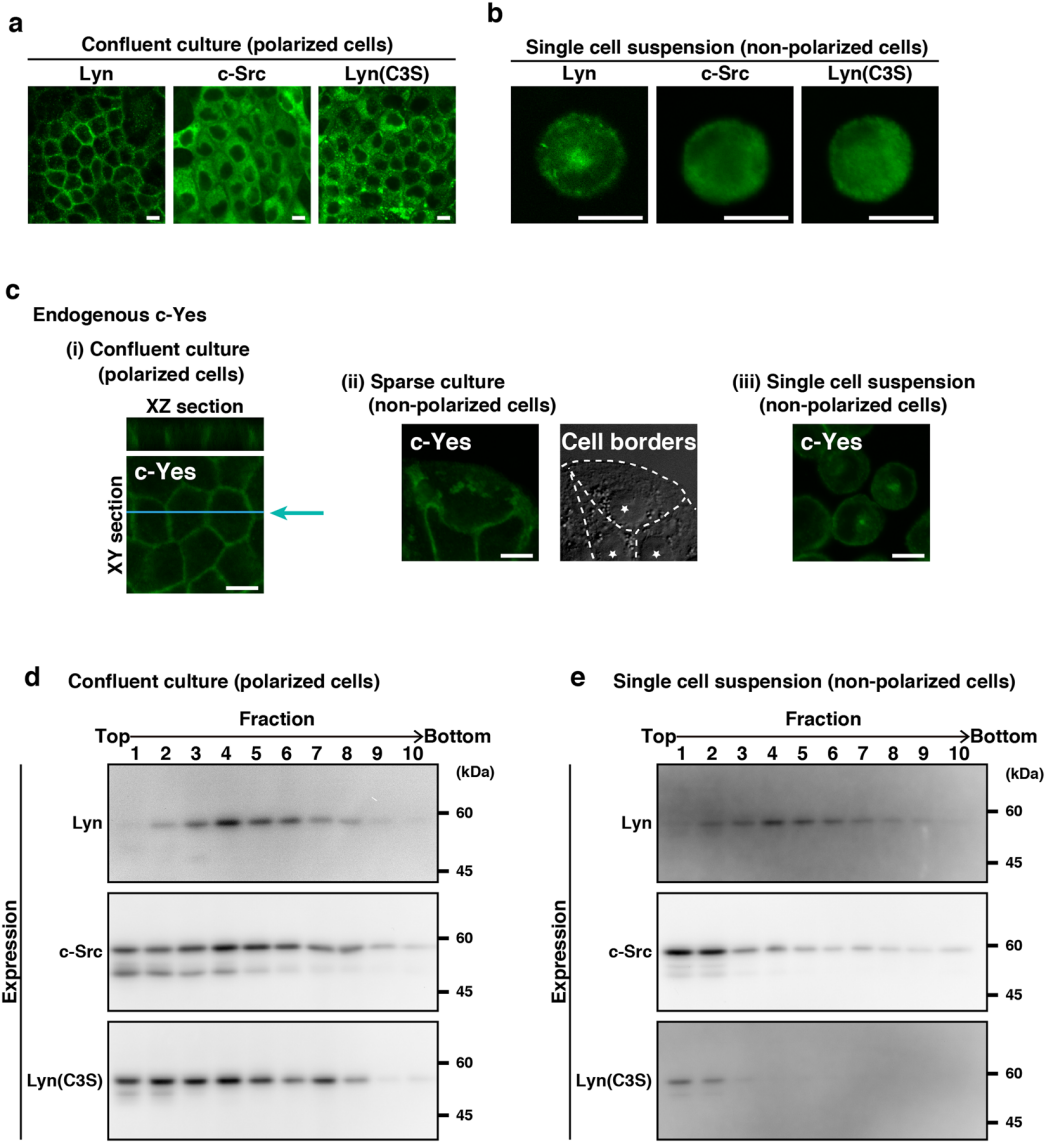
Role for palmitoylation of Lyn in its endomembrane localization in MDCK cells. **(a**,**d)** MDCK/TR/Lyn, MDCK/TR/c-Src, and MDCK/TR/Lyn(C3S) cells cultured as confluent monolayers (polarized cells) were treated with 1 μg/ml Dox for 10 h. **(b**,**e)** MDCK/TR/Lyn, MDCK/TR/c-Src, and MDCK/TR/Lyn(C3S) cells cultured to confluence (polarized cells) were treated with 1 μg/ml Dox for 8 h, detached with trypsin and subsequently cultured in spinner flasks for 2 h. **(a**,**b)** Cells were stained with mouse monoclonal anti-Lyn or anti-c-Src antibody. Scale bars, 10 μm. **(c)** Parental MDCK cells were cultured to confluence (**i**, polarized cells), cultured sparsely (**ii**, non-polarized cells), or cultured to confluence and subsequently cultured in suspension for 2 h (**iii**, single cell suspension cultures). Cells were stained with anti-c-Yes antibody. For eye guide, cell borders and nuclei are marked by dotted lines and stars, respectively **(ii)**. Scale bars, 10 μm. **(d**,**e)** Post-nuclear supernatants obtained from polarized **(d)** or single cell suspension-cultured, non-polarized **(e)** MDCK cells were subjected to sucrose density gradient fractionation, and each fraction was analysed by Western blotting with the indicated antibodies. Molecular size markers are shown in kDa. The full-length blots are presented in Supplementary Fig. S8.

Our previous study demonstrated that palmitoylation at the N-terminal region of Lyn determines its localization in HeLa and COS-1 cells^9^. To examine whether palmitoylation was required for the regulation of Lyn localization in non-polarized and polarized MDCK cells as well, we generated MDCK cell lines expressing inducible c-Src, a non-palmitoylated member of Src-family kinases, or inducible Lyn(C3S), a non-palmitoylated Lyn mutant (Cys → Ser mutation at position 3) as with inducible c-Src. In polarized MDCK cells, c-Src and Lyn(C3S) were widely distributed throughout the cell interior, whereas Lyn was largely localized to the plasma membrane (Fig. 5a). Unlike mono-palmitoylated Lyn, non-palmitoylated c-Src and Lyn(C3S) were not accumulated at the perinuclear region during a single cell suspension culture (Fig. 5b). Endogenous c-Yes, another mono-palmitoylated Src-family member, was indeed localized to the plasma membrane in polarized MDCK cells and accumulated to the perinuclear region in non-polarized MDCK cells (sparse culture or single cell suspension culture) (Fig. 5c). These results suggest that palmitoylation plays a critical role in the regulation of Lyn localization between polarization and depolarization of MDCK cells.

Furthermore, our recent study showed that the distribution pattern of Lyn in sucrose density gradient fractionation is changed by its palmitoylation state in HeLa cells^11^. We therefore compared the distribution patterns of Lyn, c-Src, and Lyn(C3S) in MDCK cells cultured as a confluent monolayer (polarized cell culture) or in a spinner flask (single cell suspension culture). In polarized MDCK cells, the majority of Lyn were distributed to Fr. 3~6, but c-Src and Lyn(C3S) were distributed throughout Fr. 1~10 (Fig. 5d). Interestingly, in single cell suspension cultures, the majority of c-Src and Lyn(C3S) became distributed to Fr. 1~2, although the distribution pattern of Lyn was quite similar to that in polarized cells (Fig. 5e). These results suggest that palmitoylation plays a critical role in directing Lyn toward endomembranes, in particular, distinct density membrane fractions.

### Translocation of Lyn to endomembranes by calcium deprivation

**Figure 6:**
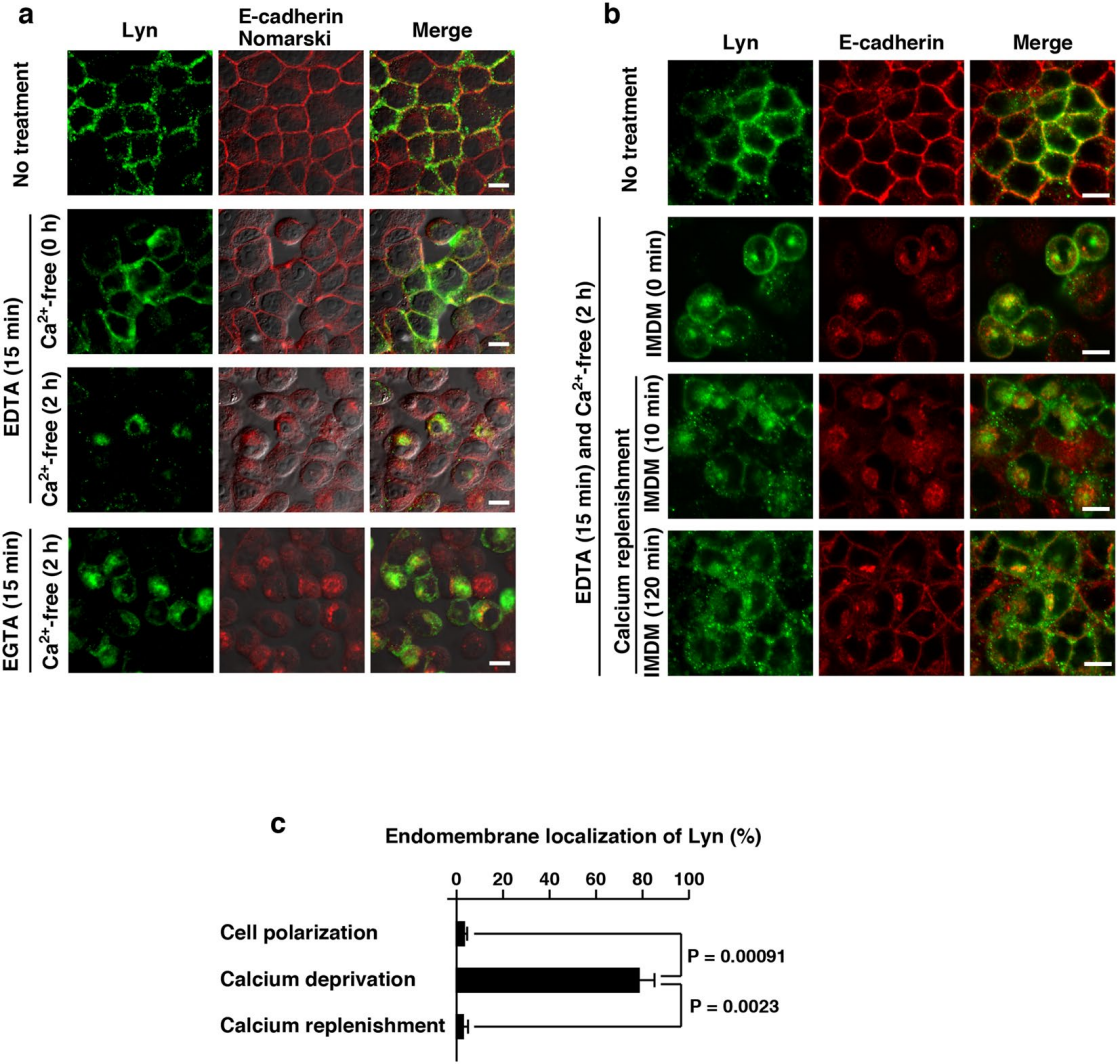
Translocation of Lyn to endomembranes by calcium deprivation. **(a)** MDCK/TR/Lyn cells cultured to confluence (polarized cells) were treated with 1 μg/ml Dox for 8 h, then treated with 4 mM EDTA or 4 mM EGTA for 15 min and subsequently cultured in calcium- and serum-free IMDM for 2 h (calcium deprivation). Cells were fixed and stained with rabbit anti-Lyn antibody (green) and anti-E cadherin antibody (red). Scale bars, 10 μm. **(b)** MDCK/TR/Lyn cells cultured to confluence (polarized cells) were treated with 1 μg/ml Dox for 8 h, then treated with 4 mM EDTA for 15 min and further incubated in calcium- and serum-free IMDM for 2 h (calcium deprivation), and subsequently incubated with IMDM containing 5% bovine serum for 10 min and 120 min (calcium replenishment). Cells were stained with anti-Lyn and anti-E-cadherin antibodies. Scale bars, 10 μm. **(c)** MDCK/TR/Lyn cells cultured to confluence (polarized cells) were treated with 1 μg/ml Dox for 8 h, then treated with 4 mM EDTA for 15 min and further incubated in calcium- and serum-free IMDM for 2 h (calcium deprivation), and subsequently incubated with IMDM containing 5% bovine serum for 20 h (calcium replenishment). Cells were stained with anti-Lyn antibody (green) and propidium iodide. The number of cells in which Lyn was predominantly accumulated to endomembranes was counted. The data represent the mean ratio ± S.D. from three independent experiments in which at least 50 cells for each category were scored. The significant differences are calculated by one-tailed Student’s *t* test.

Non-polarized MDCK cells that were sparsely cultured were still capable of partially forming cell-cell interaction sites to which Lyn was recruited (Figs 2a,c and 3a). We hypothesized that the recruitment of Lyn to cell-cell interaction sites has similar characteristics to that to the plasma membrane. Since the formation of cell-cell interactions requires extracellular calcium ions, deprivation of calcium ions by treatment of polarized MDCK cells with EDTA or EGTA breaks down cell-cell interaction sites and induces internalization of the adherence junction protein E-cadherin^25, 26^ (see Fig. 6a,b). Intriguingly, it was found that deprivation of calcium ions also induced the translocation of Lyn from the plasma membrane to endomembranes in polarized MDCK cells (Fig. 6a,b). Given that the E-cadherin trans-interactions require approximately 1 mM calcium ions^27^, IMDM medium, which contains 1.5 mM calcium ions, can supply a sufficient concentration of calcium ions to recover cell-cell interactions through E-cadherin. For 2 h of calcium replenishment after calcium deprivation, the recruitment of Lyn to the plasma membrane corresponded to the formation of calcium-dependent E-cadherin trans-interactions on cell-cell interaction sites (Fig. 6b,c). Conversely, endomembrane localization of Lyn in polarized MDCK cells was increased by calcium deprivation and decreased by calcium replenishment (Supplementary Fig. S4 and Fig. 6b,c). These results suggest that cell-cell interactions through E-cadherin are involved in the recruitment of Lyn to the plasma membrane in polarized MDCK cells.

### Relocation of Lyn to the plasma membrane by cell-cell interactions

**Figure 7:**
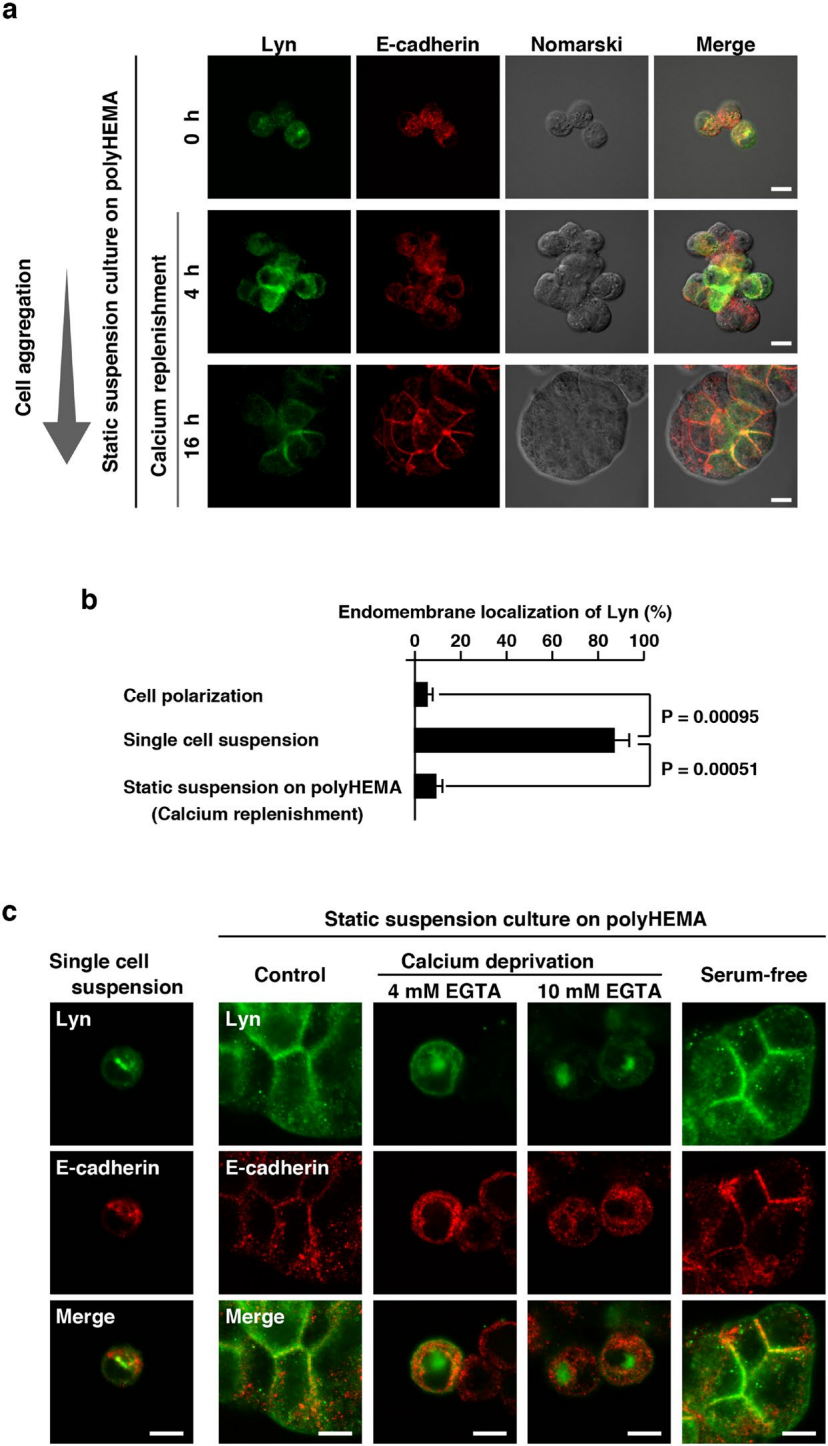
Relocation of Lyn to the plasma membrane by cell-cell interactions. **(a**,**c)** Cells cultured as described below were stained with rabbit anti-Lyn and anti-E-cadherin antibodies. Scale bars, 10 μm. **(a)** MDCK/TR/Lyn cells cultured as confluent monolayers (polarized cells) were treated with 1 μg/ml Dox for 8 h, detached by trypsinization and subsequently cultured in a spinner flask with calcium- and serum-free IMDM (single cell suspension culture). Single suspended cells were transferred into polyHEMA-coated culture dishes and cultured in IMDM-5% bovine serum (calcium replenishment) for the indicated time (static suspension culture). **(b)** Single suspended cells cultured as described in **(a)** (single cell suspension culture) were transferred into polyHEMA-coated culture dishes and cultured in IMDM-5% bovine serum (calcium replenishment and static suspension culture) for 20 h. Cells were stained with anti-Lyn antibody and propidium iodide. The number of cells exhibiting predominant accumulation of Lyn to endomembranes was counted. The data represent the mean ratio ± S.D. from three independent experiments in which at least 50 cells for each category were scored. The significant differences are calculated by one-tailed Student’s *t* test. **(c)** Cells cultured as described in **(a)** (single cell suspension culture) were transferred into polyHEMA-coated culture dishes and cultured for 16 h in IMDM containing 5% bovine serum (static suspension culture, Control), IMDM containing 5% bovine serum in the presence of 4 mM or 10 mM EGTA (static suspension culture, Calcium deprivation), or serum-free IMDM (static suspension culture, Serum-free). Scale bars, 10 μm.

It is evident that adjacent MDCK cells grown as confluent monolayers inevitably adhere to each other, and that monolayered MDCK cells also adhere to the surface of culture dishes where extracellular matrix scaffolds are associated. To examine whether cell-scaffold interactions were required for plasma membrane localization of Lyn, we transferred single suspended cells into poly(2-hydroxyethyl methacrylate) [polyHEMA]-coated culture dishes. Cells cultured on the polyHEMA-coated surface of culture dishes are well known to prevent cell adhesion to the surface of culture dishes in static suspension cultures, resulting in the formation of cell aggregates or spheroids^28^ (see Fig. 7). Single suspended cells became aggregated in a time-dependent manner during static suspension cultures, and the localizations of E-cadherin and Lyn were changed from endomembranes to the cell-cell interaction sites at the plasma membrane (Fig. 7a,b and Supplementary Fig. S5). However, calcium deprivation by EGTA treatment, but not serum-free cultures, did indeed inhibit the relocation of Lyn from endomembranes to the plasma membrane in static suspension cultures (Fig. 7c). These results suggest that calcium-dependent cell-cell interactions, even without cell-scaffold interactions, can relocate Lyn from endomembranes to the plasma membrane.

## Discussion

In the present study, we show that calcium-dependent cell-cell interactions dynamically promote plasma membrane-localization of Lyn, a mono-myristoylated and mono-palmitoylated member of Src-family kinases, in polarized MDCK cells. Interactions between adjacent MDCK cells recruit Lyn from endomembranes to the plasma membrane even without cell attachment to extracellular matrix scaffolds, and loss of cell-cell interactions by calcium deprivation relocates Lyn from the plasma membrane to endomembranes possibly through the interaction of Lyn trafficking with Rab11-mediated endosomal trafficking.

Polarized membrane trafficking in cells is intertwined with various biological functions, including migration, differentiation, cell division, and epithelial barrier formation^12, 14^. MDCK cell monolayers can form apical-basal cell polarity, which is widely used as a model system for studying epithelial polarity. Previous studies showed that c-Src, Lyn and c-Yes, three members of Src-family kinases, all localize to the plasma membrane in primary cultured rat liver cells and an epithelial cell line derived from the kidney^3^. At the plasma membrane of polarized MDCK cells, Src-family kinases are involved in stabilization of intercellular adhesions and activation of transcytosis^16, 17^. Generation of an MDCK cell clone that equips with a tetracycline-inducible protein expression system successfully ensures inducible expression of exogenous human Lyn at any time irrespective of polarization (Fig. 1). Use of our MDCK cell line expressing inducible Lyn enables us to scrutinize the relationship between membrane trafficking and polarization. We thus reveal that Lyn is not statically localized to the plasma membrane but is distributed between the plasma membrane and endomembranes in a manner dependent on either polarization or depolarization.

Our previous studies showed several characteristics of the translocation of Lyn in COS-1 and HeLa cells: (i) biosynthetic trafficking of Lyn to the plasma membrane via the Golgi region^8^, (ii) palmitoylation-dependent differential trafficking of Lyn^9, 21^, and (iii) biochemical changes in Lyn distribution in sucrose density gradient fractionation upon cell detachment^11^. In this study, we grew MDCK cells in various culture conditions and examined the relationship between membrane trafficking and cell polarity. We cultured MDCK cells under the following five different conditions: (i) cells were cultured to confluence until they form tight junctions, thereby being fully polarized in monolayers (Fig. 2), (ii) cells were sparsely cultured, where cells were contacted on not all sides with adjacent cells (adherent, non-polarized cells) (Fig. 2a), (iii) suspended cells were grown in a spinner flask, so that they became round in shape and were depolarized during a single cell suspension culture (Figs 3–5), (iv) polarized cells are treated with EDTA or EGTA, which can still adhere to extracellular matrix scaffolds (adherent) but lose intercellular adhesions (Fig. 6), and (v) suspended cells were cultured in polyHEMA-coated dishes, so that cells are incapable of adhering to extracellular matrix scaffolds (non-adherent) but retain the ability to induce intercellular adhesions/cell aggregation (Fig. 7). Accordingly, we can reveal the following evidence: (i) differential localizations of Lyn in polarized and non-polarized MDCK cells are sustained for at least 30 h after induction of Lyn expression (Fig. 3a), (ii) palmitoylation is critical for Lyn localization in MDCK cells (Fig. 5a,b), (iii) cell detachment does not change Lyn distribution in sucrose density gradient fractionation (Fig. 5d,e), (iv) Lyn localization is appreciably changed from the plasma membrane to endomembranes by suppression of intercellular adhesions by calcium deprivation (Fig. 6), and (v) Lyn is reversibly accumulated to the plasma membrane by intercellular re-adhesions even without cell attachment to extracellular matrix scaffolds (Fig. 7). Taken together, these results suggest that intercellular adhesions play a critical role in Lyn localization to the plasma membrane in polarized MDCK cells. Unlike Lyn, c-Src did not show appreciable changes in its staining pattern (Fig. 5a,b). However, the distribution of c-Src in membrane fractionation was changed upon cell depolarization (Fig. 5d,e). These results implicate an additional mechanism for explaining the differences of Src-family kinases in density-membrane fractionation during cell polarization.

The signal transduction pathways responding to the tension from adjacent cells and the stiffness of extracellular matrix scaffolds are important for cellular functions^29, 30^. Single cell suspension culture is an appropriate method to dissociate cell-cell interactions and minimize the stiffness of extracellular matrix scaffolds. Localization of Src-family kinases in single suspended cells has been thus far examined with at least three cell lines, such as HeLa, NIH3T3, and MDCK cells. Our present study shows that cell-cell interactions are important for the regulation of Lyn translocation from endomembranes to the plasma membrane in polarized MDCK cells. Meanwhile, in HeLa cells, cell detachment changes Lyn distribution in sucrose density gradient fractionation but does not internalize Lyn from the plasma membrane^11^. In NIH3T3 cells, dissociation of cell-scaffold interactions induces the translocation of Src-family kinases from the plasma membrane to endomembranes^31^. Considering that Src-family kinases located at the plasma membrane are involved in both cell-cell interactions and cell-scaffold interactions^32^, we hypothesize that the localization of Src-family kinases to the plasma membrane may be differentially regulated in respective cell conditions.

Once polarized MDCK cells lose intercellular adhesions, Lyn and E-cadherin become accumulated from the plasma membrane to endomembranes (Figs 6 and 7). It is known that internalized E-cadherin is recruited again to the plasma membrane via recycling endosomes or degraded in lysosomes^33^, and that active Rab11 is required for the trafficking of E-cadherin from recycling endosomes to the plasma membrane in polarized MDCK cells^34^. Rab11 regulates not only exocytic recycling but also inter-endosomal trafficking from recycling endosomes^22, 24^. For example, Rab11 is required for compartmentalization of GLUT4 to storage vesicles under insulin-starved conditions^35^. In single suspended, non-polarized MDCK cells, endosomal Lyn does co-localize even partially with the recycling endosome marker Rab11 but does not overlap with the lysosome marker cathepsin D (Fig. 4b). Overexpression of GDP-locked Rab11(S25N) dominant-negatively perturbs perinuclear accumulation of Lyn in a single cell suspension culture (Fig. 4c), suggesting a direct or indirect interaction of active Rab11 with endosomal trafficking of Lyn. Note that Lyn does not co-localize with E-cadherin on endomembranes in single suspended cells (Fig. 7c). These results suggest that the trafficking pathway of Lyn may be different from that of E-cadherin in depolarized MDCK cells. Thus, we hypothesize that, upon loss of cell-cell interactions, MDCK cells may form a particular endomembrane compartment that accumulates Lyn in a Rab11-dependent manner.

In conclusion, using our established MDCK cell line that can inducibly express a protein of interest in a polarized state, we show for the first time that calcium-dependent cell-cell interactions play a critical role in Lyn localization at the plasma membrane in polarized MDCK cells. Further exploration with MDCK cell lines expressing inducible proteins of interest will help us to understand the dynamic pathways for the trafficking of apical and basolateral proteins during polarization and depolarization.

## Methods

### Plasmids

cDNAs encoding human Lyn^36^ (the 56-kDa isoform) (provided by Tadashi Yamamoto) and human c-Src^37^ (provided by Donald J. Fujita) were subcloned into the pcDNA4/TOneoR vector^38^. The Cys → Ser mutation at position 3 on Lyn [Lyn(C3S)] was generated previously^11^. HA-Rab11-wt (wild-type Rab11)^39^ and HA-Rab11S25N (dominant-negative Rab11)^39^ subcloned into the pcDNA3.1 vector (Invitrogen) was provided by Stephen Ferguson. Rab5aS34N (dominant-negative Rab5a)^40^ subcloned into the pEGFP-C1 vector (Clonetech) was provided by Brian J. Knoll. Rab7N125I (dominant-negative Rab7)^41^ subcloned into the pEGFP-C1 vector was provided by Bo van Deurs. The CAG promoter-driven pCAG/TR vector expressing the tetracycline repressor (TR) was constructed previously^42^. The sequences selected for short hairpin RNA (shRNA) against canine Lyn were as follows: shLyn #1, GTCTGATGTGTGGTCCTTT; shLyn #2, GCACTACAAAATTAGAAGT. The oligonucleotides for shRNA were subcloned into the mCherry-coexpressing shRNA vector, as described^43^.

### Antibodies

The following antibodies were used: mouse monoclonal anti-Lyn (Lyn9, Wako Pure Chemicals, Osaka, Japan; 1:500 for Western blotting, 1:50 for Immunofluorescence), rabbit polyclonal anti-Lyn (GTX111584, GeneTex; 1:500 for Western blotting, 1:500 for Immunofluorescence), anti-Src (GD11, Millipore; 1:500 for Western blotting, 1:200 for Immunofluorescence), anti-actin (C4, Millipore; 1:1000 for Western blotting), anti-E-cadherin (610181, BD Transduction Laboratories; 1:1000 for Western blotting, 1:500 for Immunofluorescence), anti-EEA1 (610456, BD Transduction Laboratories; 1:200 for Immunofluorescence), anti-ZO-1 (Invitrogen; 1:500 for Immunofluorescence), anti-GM130 (610822, BD Transduction Laboratories; 1:1000 for Immunofluorescence), anti-furin (Thermo Scientific; 1:200 for Immunofluorescence), anti-TR (MoBiTec; 1:400 for Western blotting), anti-Rab11 (Invitrogen; 1:50 for Immunofluorescence), anti-HA (Y-11, Santa Cruz Biotechnology; 1:100 for Immunofluorescence), anti-Yes (clone 1, BD Transduction Laboratories; 1:200 for Immunofluorescence), and anti-cathepsin D (DakoCytomation; 1:250 for Immunofluorescence). Horseradish peroxidase-F(ab′)_2_ fragments of anti-mouse IgG antibody (Jackson ImmunoResearch) and anti-rabbit IgG antibody (Jackson ImmunoResearch) were used. Alexa Fluor 488-donkey anti-mouse IgG, Alexa Fluor 488-donkey anti-rabbit IgG, Alexa Fluor 647-donkey anti-mouse IgG, and Alexa Fluor 647-donkey anti-rabbit IgG antibodies obtained from Invitrogen were used.

### Cells and transfection

MDCK cells (provided by Toshiharu Horie) and MDCK-derived cells were cultured in Iscove’s modified Dulbecco’s medium (IMDM) containing 5% bovine serum. To generate MDCK stable cell lines for tetracycline-inducible expression of Lyn, c-Src or Lyn(C3S), MDCK cells were stably co-transfected with pCAG/TR and a plasmid containing the hygromycin resistance gene and selected in 250 μg/ml hygromycin. MDCK cells stably expressing TR (MDCK/TR cells) were transfected with pcDNA4/TO/Lyn, pcDNA4/TO/c-Src, or pcDNA4/TO/Lyn(C3S) and selected in 250 μg/ml G418. Doxycycline, a tetracycline derivative, was used at 1 μg/ml for inducible expression of Lyn, c-Src, or Lyn(C3S). Transfection was performed using linear polyethylenimine (25 kDa; Polysciences)^44^.

### Adhesion and suspension cultures

For non-polarized-cell culture, MDCK cells (1.5 × 10^4^ cells/mL; 3.1 × 10^3^ cells/cm^2^) were sparsely seeded on tissue culture dishes (Falcon) and cultured for 2 days. For polarized-cell culture, MDCK cells (1.0 × 10^5^ cells/mL; 2.1 × 10^4^ cells/cm^2^) were cultured to confluence for 2 days and further 2~4 days post-confluence. For single cell suspension cultures, cells were washed with Dulbecco’s phosphate-buffered saline (D-PBS), incubated with 0.25% trypsin for 2 min at 37 °C and then cultured in a spinner flask with calcium-deprived IMDM. For static suspension cultures, cells detached by trypsinization were transferred into poly(2-hydroxyethyl methacrylate) [polyHEMA]-coated culture dishes and cultured in IMDM containing 5% bovine serum. PolyHEMA-coated dishes were prepared as described recently^11^. In brief, tissue culture dishes were filled with 3% (w/v) polyHEMA (Sigma) dissolved in 95% ethanol, and then ethanol was evaporated under air blow for 1 h.

### Immunofluorescence microscopy

Immunofluorescence staining was performed as described recently^11^. In brief, cells were fixed in PBS-containing 2% paraformaldehyde for 20 min at 37 °C, permeabilized with 0.1% Triton X-100 for 3 min at room temperature, blocked in PBS containing 3% bovine serum albumin and 0.1% saponin, and then sequentially incubated with a primary and a secondary antibody for 1 hour each. For endogenous Lyn staining, cells were fixed and permeabilized in 100% methanol at −20 °C for 10 min. For staining of nuclei, cells were treated with 200 μg/mL RNase A and 10 μg/mL propidium iodide for 30 min. Confocal and Nomarski differential-interference-contrast images were acquired at 512 × 512 pixels using a FluoView FV500 laser scanning microscope with a 60x water-immersion objective lens (Olympus, Tokyo, Japan). One-planer (xy) section slice images with 0.6-μm thickness were shown. Orthogonal sections viewing axial directions (xz) were created when all Z-series sections at 0.35 μm intervals were merged. Composite figures were prepared using FV10-ASW version 2 viewer, GIMP 2.6.4 software, and Adobe Illustrator CS6. The Pearson’s R values were computed using Fiji software.

### Western blotting

Cell lysates prepared in SDS-sample buffer containing the protein tyrosine phosphatase inhibitor Na_3_VO_4_ (10 mM) were separated by SDS-PAGE and electro-transferred onto polyvinylidene difluoride membranes (PVDF, Millipore), as described^45^. Immunodetection was performed as reported previously^11^. Results were analysed using an image analyser ChemiDoc XRSplus (Bio-Rad). Intensity of chemiluminescence was measured using the Quantity One software (Bio-Rad). Images were cropped and labelled using GIMP 2.6.4 software and Adobe Illustrator CS6.

### Sucrose density gradient fractionation

Sucrose density gradient fractionation was performed as described previously^11, 20^. In brief, cells were swollen in hypotonic buffer (40 mM HEPES, pH 7.4, 1 mM MgSO_4_, and 10 mM Na_3_VO_4_) containing protease inhibitors, followed by homogenization with 20 strokes of a tight-fitting 1-mL Dounce homogenizer. After adjusting the sucrose concentration to 250 mM, the post-nuclear supernatants were recovered by centrifugation at 1,000 × *g* for 5 min. The resultant post-nuclear supernatants (900 μl) were loaded at the top of a discontinuous sucrose gradient, composed of successive layers of 800 μl each of hypotonic buffer containing 1.5 M, 1.2 M, 1.0 M, 0.8 M, and 0.5 M sucrose. After centrifugation at 100,000 × *g* for 85 min in a P55ST2 rotor (Hitachi, Tokyo, Japan), 10 fractions of 490 μl were collected from the top of the tube and analysed by Western blotting. All steps were carried out at 4 °C.

## Electronic supplementary material

Supplementary Information
