# Supplementary material for: Recruitment of Lyn from endomembranes to the plasma membrane through calcium-dependent cell-cell interactions upon polarization of inducible Lyn-expressing MDCK cells
Source: Sci Rep. 2017 Mar 28;7:493. doi: 10.1038/s41598-017-00538-5 (PMC5428707; doi:10.1038/s41598-017-00538-5)
Supplement: Supplementary file 1 — Supplementary Information [file 41598_2017_538_MOESM1_ESM.pdf]

Supplementary information

Recruitment of Lyn from endomembranes to the plasma membrane through calcium-dependent cell-cell interactions upon polarization of inducible Lyn-expressing MDCK cells

Takao Morinaga[1,2], Sayuri Yanase[1], Aya Okamoto[1], Noritaka Yamaguchi[1], and Naoto Yamaguchi[1,*]

[1]Laboratory of Molecular Cell Biology, Graduate School of Pharmaceutical Sciences, Chiba University, Chiba 260-8675, Japan
[2]Division of Pathology and Cell Therapy, Chiba Cancer Center Research Institute, Chiba 260-8717, Japan

*To whom correspondence should be addressed: Naoto Yamaguchi, Ph.D. Laboratory of Molecular Cell Biology, Graduate School of Pharmaceutical Sciences, Chiba University, Inohana 1-8-1, Chuo-ku, Chiba 260-8675, Japan. Phone & Fax: +81-43-226-2868. E-mail: nyama@faculty.chiba-u.jp

Supplementary Figure S1
Comparison of the antibody reactivity between human and canine Lyn.

Supplementary Figure S2
Localization of ZO-1 in polarized MDCK/TR/Lyn cells expressing inducible Lyn.

Supplementary Figure S3
Knockdown of endogenous Lyn in non-polarized MDCK cells.

Supplementary Figure S4
Localization of Lyn in polarized MDCK cells upon calcium deprivation and replenishment.

Supplementary Figure S5
Comparison of Lyn localization between single cell suspension cultures and static suspension cultures.

Supplementary Figure S6
The full-length blots for Figure 1.

Supplementary Figure S7
The full-length blots for Figure 3c.

Supplementary Figure S8
The full-length blots for Figure 5.

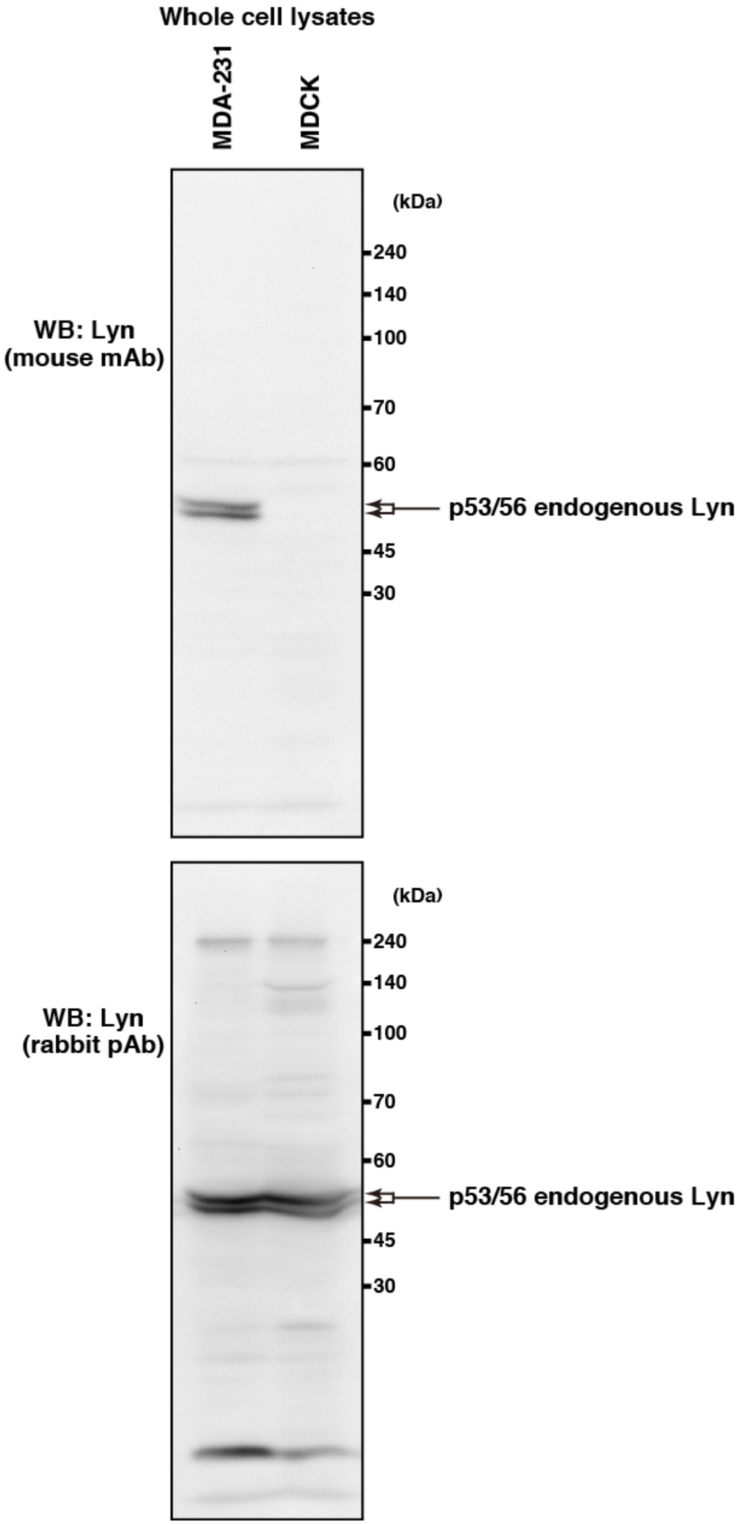

Supplementary Figure S1.

Comparison of the antibody reactivity between human and canine Lyn.

Whole cell lysates obtained from the human epithelial cell line MDA-MB-231 and the canine epithelial cell line MDCK were subjected to Western blot analysis with mouse monoclonal anti-Lyn antibody (mAb) and rabbit polyclonal anti-Lyn antibody (pAb).

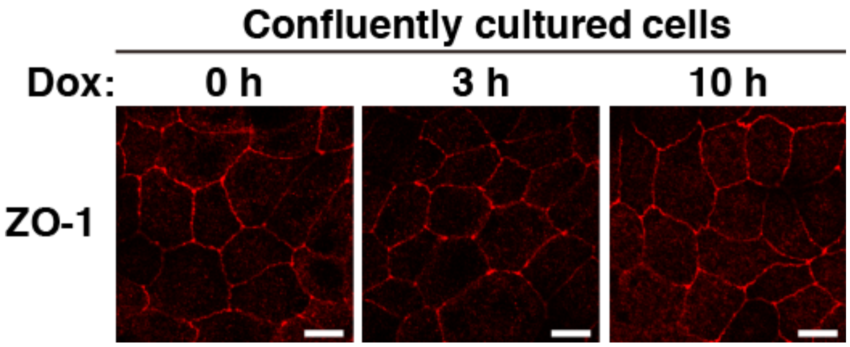

Supplementary Figure S2.

Localization of ZO-1 in polarized MDCK/TR/Lyn cells expressing inducible Lyn.

MDCK/TR/Lyn cells cultured to confluence (polarized cells) were treated with 1 µg/ml Dox for the indicated time (the level of Lyn expression at each time point is shown in Fig. 1). Cells were fixed and stained with anti-ZO-1 antibody (red). Representative data were shown. Scale bars, 10 µm.

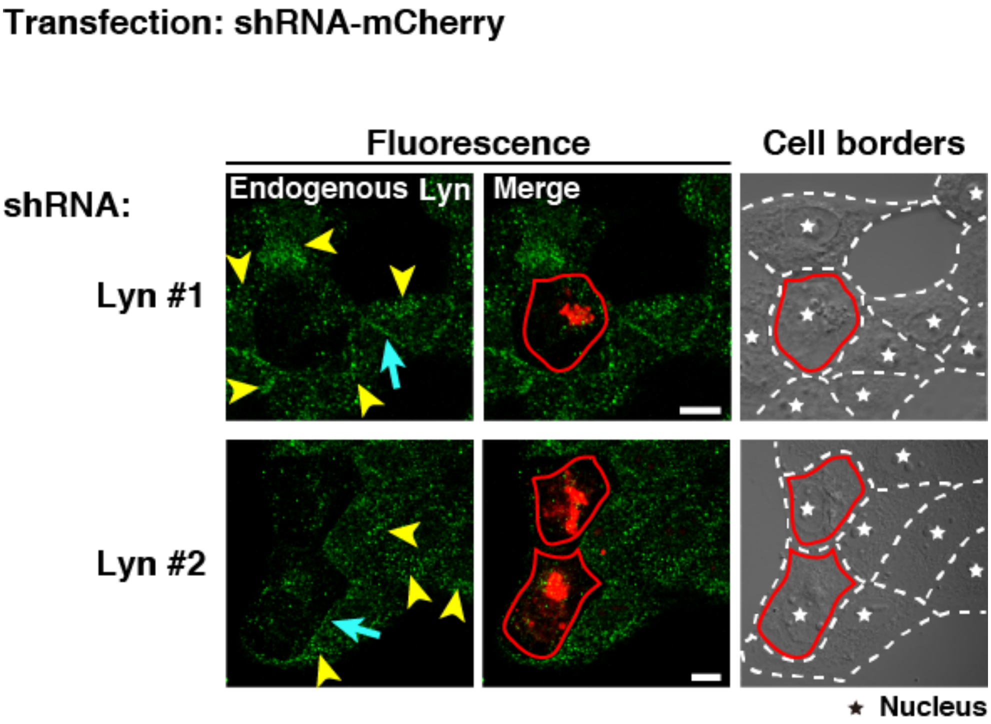

Supplementary Figure S3.

Knockdown of endogenous Lyn in non-polarized MDCK cells.

Sparsely cultured MDCK cells transfected with shRNA-mCherry vector targeting two different sequences on Lyn mRNA (Lyn #1 and Lyn#2) were cultured for 60 h. Endogenous Lyn and mCherry were visualized with rabbit polyclonal anti-Lyn antibody (green) and mCherry fluorescence (red), respectively. Cells expressing mCherry are indicative of cells co-expressing shLyn (cells marked in red). Note that the staining of endogenous Lyn was specifically diminished in cells expressing mCherry. For eye guide, cell borders and nuclei were marked by dotted lines and stars, respectively. Arrows (blue) indicate plasma membrane-localized endogenous Lyn; arrowheads (yellow) indicate endomembrane-localized endogenous Lyn. Scale bar, 10 µm.

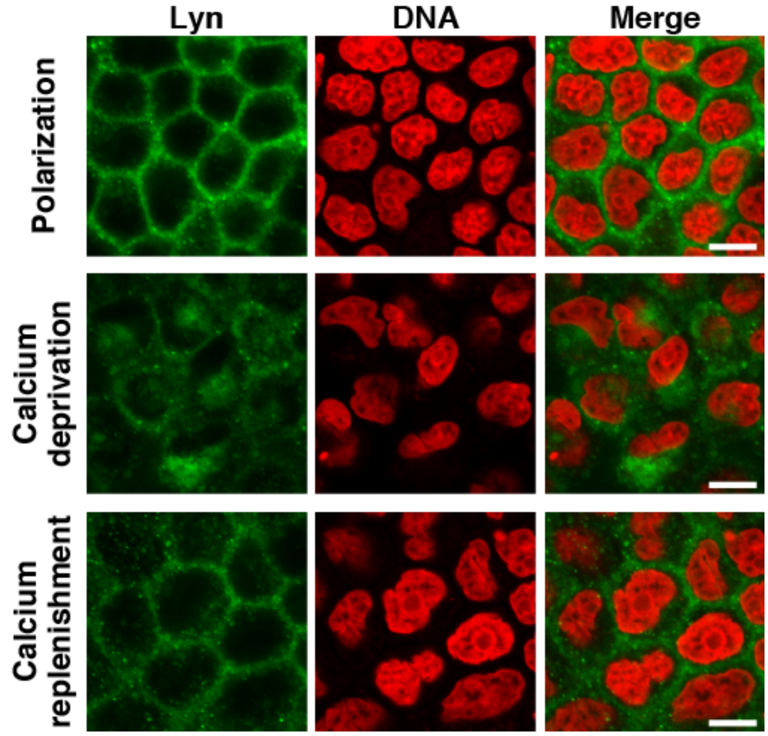

Supplementary Figure S4.

Localization of Lyn in polarized MDCK cells upon calcium deprivation and replenishment.

MDCK/TR/Lyn cells cultured to confluence (polarized cells) were treated with 1 μg/ml Dox for 8 h, then treated with 4 mM EGTA for 15 min, cultured in calcium- and serum-free IMDM for 2 h (calcium deprivation), and subsequently incubated with IMDM containing 5% bovine serum for 20 h (calcium replenishment). Cells were fixed and stained with anti-Lyn antibody (green) and propidium iodide (red). Representative data were shown. Scale bars, 10 μm.

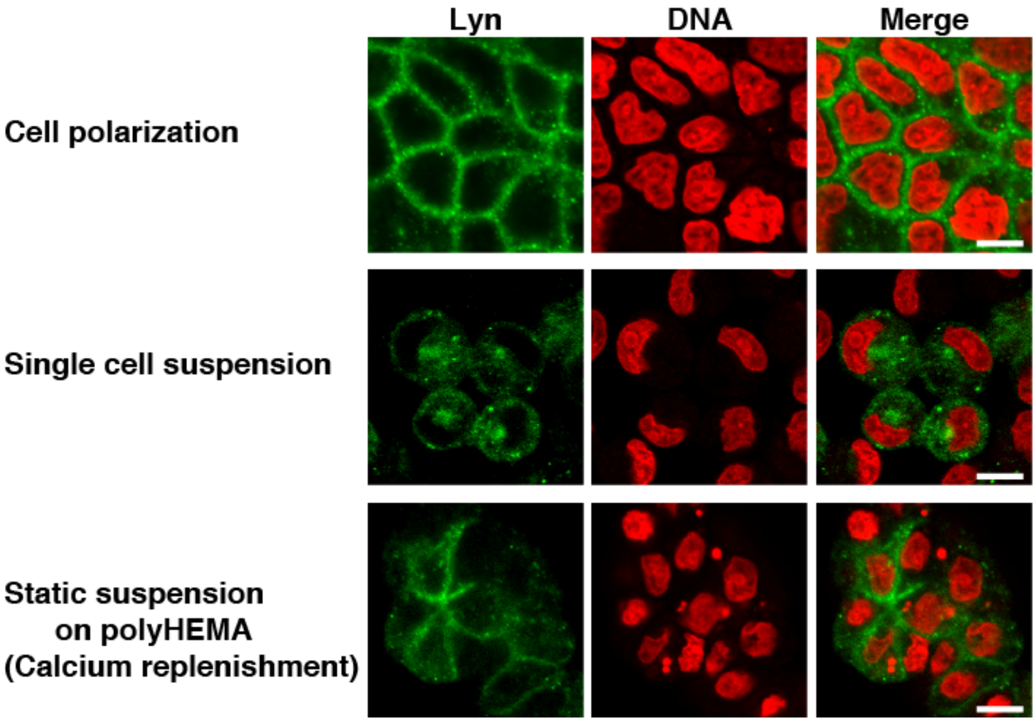

Supplementary Figure S5.

Comparison of Lyn localization between single cell suspension cultures and static suspension cultures.

MDCK/TR/Lyn cells cultured as confluent monolayers (polarized cells) were treated with 1 μg/ml Dox for 8 h, detached by trypsinization and subsequently cultured in a spinner flask with calcium- and serum-free IMDM (single cell suspension cultures). Single suspended cells were transferred into polyHEMA-coated culture dishes and cultured in IMDM-5% bovine serum for 20 h (calcium replenishment and static suspension cultures). Cells were fixed and stained with anti-Lyn antibody (green) and propidium iodide (red). Representative data were shown. Scale bars, 10 μm.

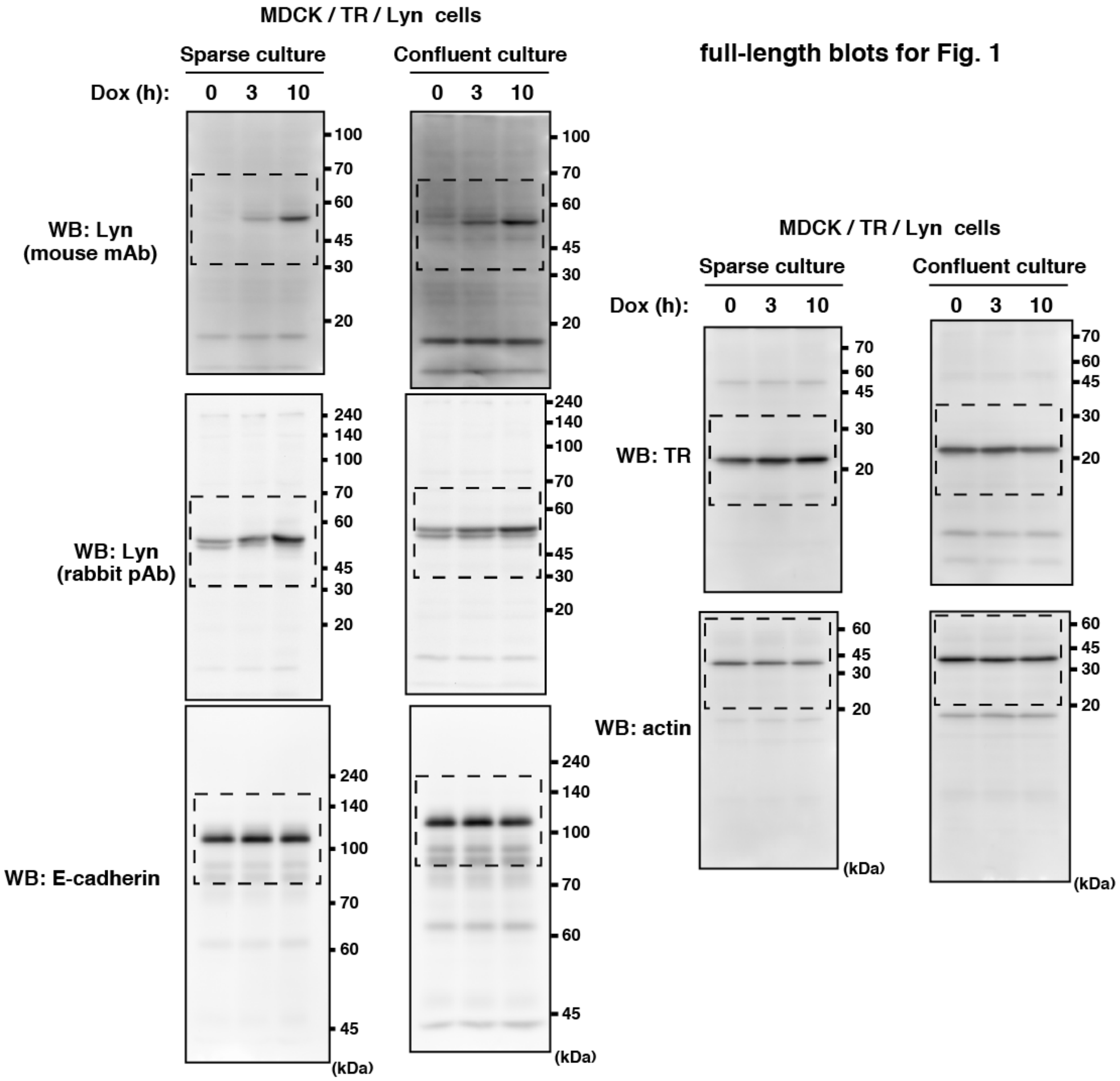

Supplementary Figure S6.

The full-length blots for Figure 1.

Dashed line boxes indicate the cropped images used in Fig. 1.

full-length blots for Fig. 3c

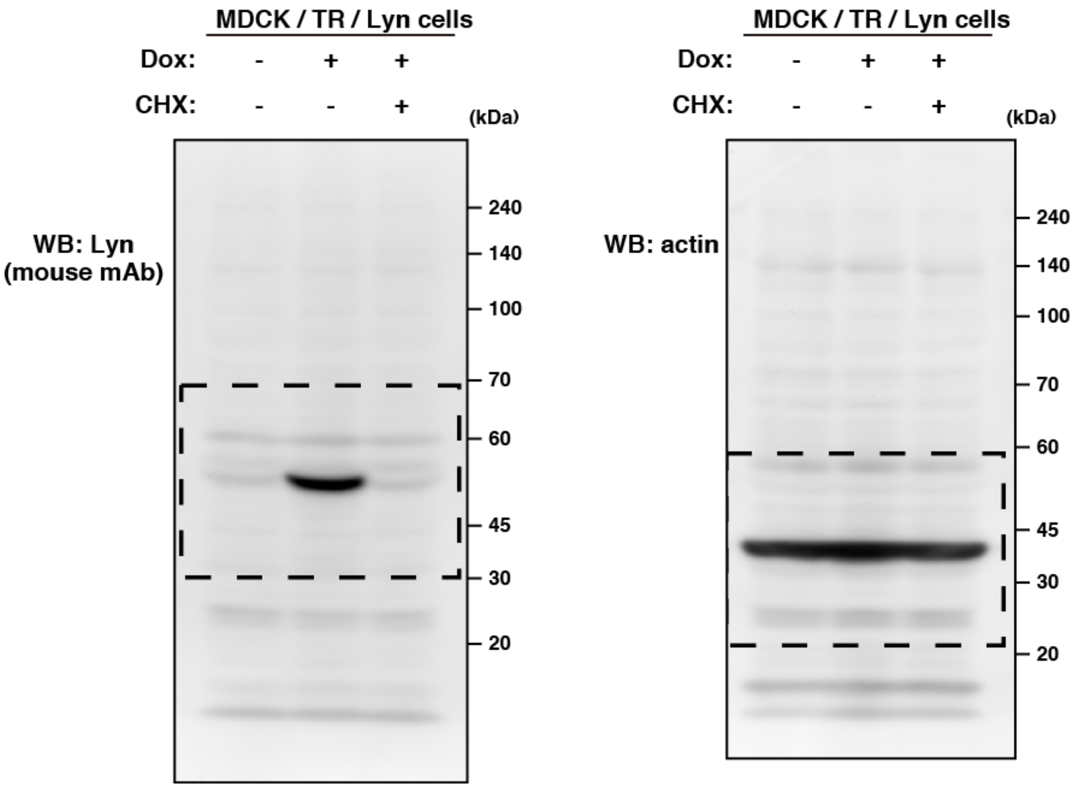

Supplementary Figure S7.

The full-length blots for Figure 3c.

Dashed line boxes indicate the cropped images used in Fig. 3c.

full-length blots for Fig. 5

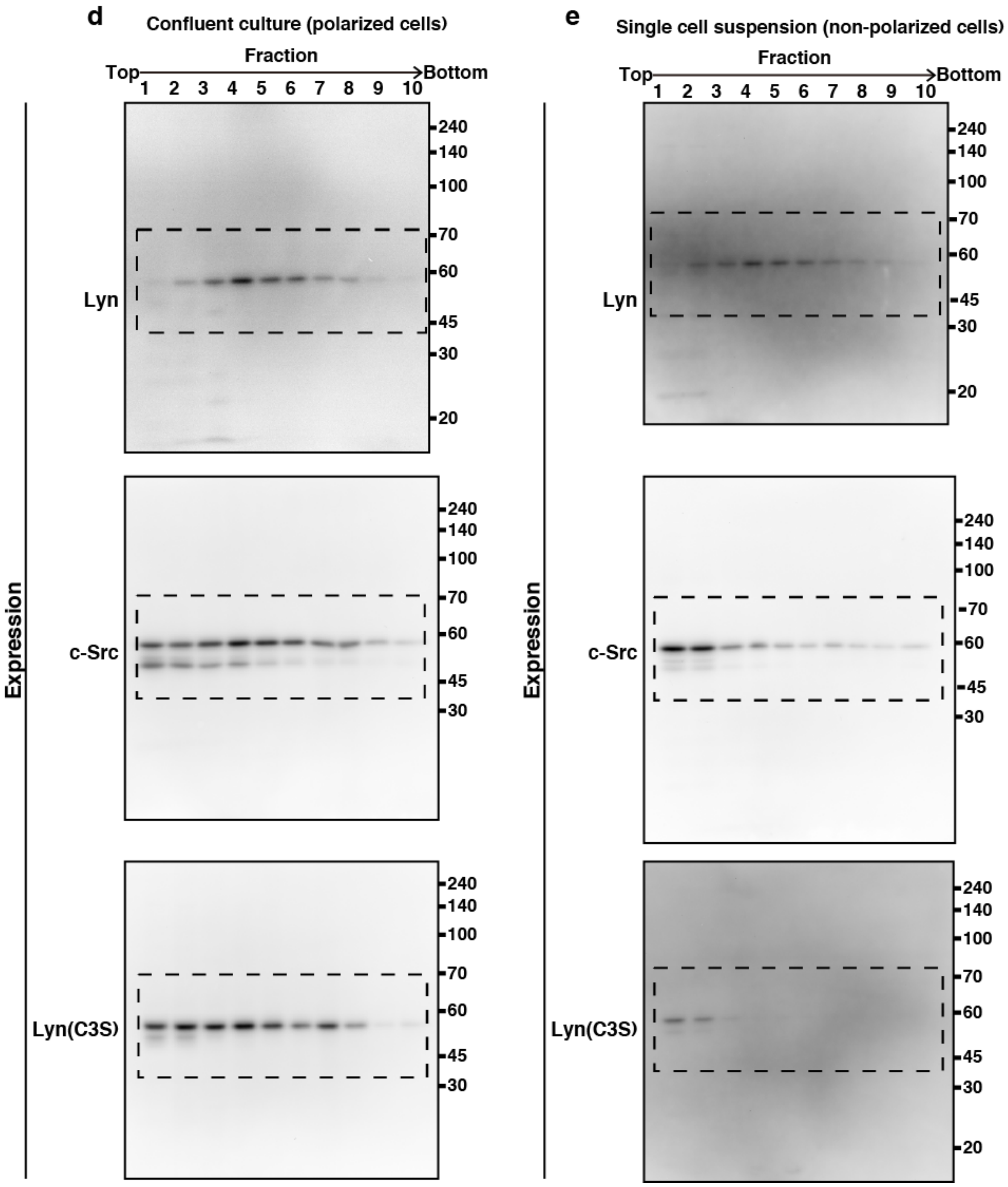

Supplementary Figure S8.

The full-length blots for Figure 5.

Dashed line boxes indicate the cropped images used in Fig. 5.
